# Clinical and Cost Burden of Primary Sjögren’s Syndrome: Descriptive Analysis Using a US Administrative Claims Database

**DOI:** 10.36469/9807

**Published:** 2018-02-22

**Authors:** Sue Perera, Liyuan Ma, Raj Punwaney, Sulabha Ramachandran

**Affiliations:** 1 GSK, Uxbridge, London, UK; 2 GSK, Pennsylvania, PA, USA; 3 GSK, King of Prussia, PA, USA

**Keywords:** treatment patterns, sjögren’s syndrome, sicca syndrome, healthcare resource utilization, healthcare administrative claims, economic burden of disease, dryness symptoms

## Abstract

**Background:** Current knowledge of the disease burden of primary Sjögren’s syndrome (pSS) is limited.

**Objectives:** The primary objective of this study was to describe the demographic and clinical characteristics of patients with pSS. The secondary objective was to describe the treatment patterns and healthcare resource utilization of patients with pSS. Furthermore, clinical characteristics of interest were described and the proportions of patients with glandular versus extra-glandular disease were reported.

**Methods:** This was a retrospective cohort study (HO-15-16077) conducted in the US Truven MarketScan Commercial database. We report descriptive data from employees and their families, as covered by employer-sponsored medical insurance. The primary cohort comprised patients with pSS (with ≥1 diagnosis of sicca syndrome prior to January 1, 2013), with continuous enrollment for ≥24 months (January 1, 2012–December 31, 2013). Patients with conditions mimicking sicca symptoms not due to SS were excluded, as were those with connective tissue disease that may have suggested secondary SS. To compare the healthcare burden of patients with and without sicca symptoms, a 1:1 matched comparator population comprising subjects without a previous diagnosis of sicca syndrome (sicca-free) was also identified.

**Results:** There were 12 717 eligible patients in the primary cohort. The majority (86%) was female and the mean age was 51 years. Overall, 60.7% of patients had claims associated with pSS extra glandular disease manifestations. These patients experienced a higher disease burden, and more commonly reported pain, fatigue or insomnia and any combination of pain, fatigue or insomnia (41.3%) compared with patients with glandular disease only (12.4%). Patients in the primary cohort incurred greater annual healthcare service costs (1.6 times greater, all causes) and healthcare resource utilization compared with the sicca-free comparator cohort. Patients with extra glandular disease also incurred greater average annual costs (2.9 times) contributing to ≥2 times/year more resource use for outpatient services than patients with glandular disease only.

**Conclusion:** Patients with pSS experience a high disease burden despite treatment. This study provides novel insights in to the extent of the burden on healthcare resources among patients with pSS, in particular for patients with extra-glandular disease manifestations, when compared with sicca-free subjects.

| This is an Open Access article under the CC BY 4.0 license.

## INTRODUCTION

Sjögren's syndrome (SS) is a chronic, autoimmune disorder primarily characterized by inflammation of the exocrine glands.[1] SS typically causes functional impairment of the salivary and lacrimal glands, presenting as dryness of the mouth and eyes ('sicca syndrome'), and is often associated with fatigue and pain. Primary SS (pSS), in contrast to secondary SS, occurs in the absence of other underlying autoimmune conditions including rheumatoid arthritis, systemic lupus erythematosus, or scleroderma. In addition to inflammation of the exocrine glands, around half of the patient population report neurological, pulmonary and renal problems, and vasculitis.[2-5] Long-term follow-up studies (5 to 12 years duration) have demonstrated that, due to the slow progression of the disease, patients with exocrine inflammation only, and those with exocrine inflammation as well as systemic manifestations, retain the serologic and clinical state with which they initially presented, with no additional clinical worsening.[6,7]

Although the precise etiology of pSS is not fully understood, dysregulation of the innate and adaptive immune systems is an important factor.[1-8] Evidence suggests that B cells play an integral role in the pathogenesis of pSS, with patients exhibiting an altered distribution of B cell subsets.[9-12] This disrupted B cell profile may be caused by elevated levels of the B cell stimulating factor (BLyS) that regulates B cell proliferation.[13] BLyS levels have been positively correlated with titers of the autoreactive antibodies that are characteristic of pSS.[14]

Previous studies indicate an average population prevalence of pSS ranging from 0.03% to 2.7%; difficulties in detection and the lack of standardized criteria for classifying the disorder likely contribute to the wide range of prevalence figures.[15] pSS is more commonly seen in females, with female to male ratios varying from 20:1 to 9:1 across different studies.[15] Although the reason for this imbalance is not fully understood, a link between androgen deficiency in women and a regulatory role of androgens on the lacrimal gland has been proposed.[15-17] pSS is also more prevalent in Caucasian adults,[15] with the age of onset typically in the fourth to fifth decade.[15] While there is limited information on the disease burden of pSS, a number of studies have demonstrated that pSS represents a significant burden to the patient (both financially and in terms of quality of life) as well as to the healthcare system due to the high use of resources such as hospital visits and diagnostic tests.[18-20] Truven databases include some of the largest healthcare claims databases in the US, providing employer-based patient data reflecting real-world treatment patterns and costs.

This retrospective cohort study therefore aimed to provide an initial exploratory descriptive analysis of the clinical presentation and disease burden of patients with pSS, describe treatment patterns, medication use, and define the healthcare resource use and costs associated with these patients, by analyzing the US Truven MarketScan databases.

## METHODS

### Study design and data source

This was a descriptive, retrospective cohort study (GSK study HO-15-16077) conducted in three US Truven MarketScan databases (Commercial, Medicare Supplemental, and Medicaid). The Medicare Supplemental and Medicaid databases were not the focus of this analysis as they provide a smaller sample size that is less representative of the general US population compared with the Commercial database, and data are more difficult to interpret given the inherent aspects of the populations captured. Here we report results from the Commercial database, which comprises medical and drug data from employers and health plans, encompassing employees, their spouses and children, as covered by employer-sponsored medical insurance. The protocol for this study was approved by the Health Outcomes Departmental Protocol Review Committee.

## Objectives

The primary objective of this study was to describe the demographic and clinical characteristics of patients with pSS. The secondary objective was to describe the treatment patterns and healthcare resource utilization of patients with pSS.

## Study population

There is no single International Classification of Diseases – 9th edition (ICD-9) diagnosis code available for SS. Therefore, to be eligible for the analysis, patients must have had at least one diagnosis of sicca syndrome (ICD 710.2), a characteristic feature of pSS, made at any point between January 1, 2003 and January 1, 2013 (Figure 1). Continuous enrollment for a minimum period of 24 months between January 1, 2012 and December 31, 2013 ensured sufficient medical history to apply the exclusion criteria. Patients with any record of head and neck radiation, Hepatitis C, acquired immune deficiency syndrome, sarcoidosis, graft-versus-host disease, amyloidosis, or immunoglobulin G4-related diseases were excluded from the analysis as they may present with sicca symptoms not due to pSS. Additionally, patients with connective tissue diseases such as rheumatoid arthritis, systemic lupus erythematosus, and scleroderma were excluded as these are suggestive of secondary SS. Not all autoimmune connective tissue diseases were considered for exclusion to avoid adding extra complexity to the selection criteria.

**Figure 1.** Primary Cohort Eligibility

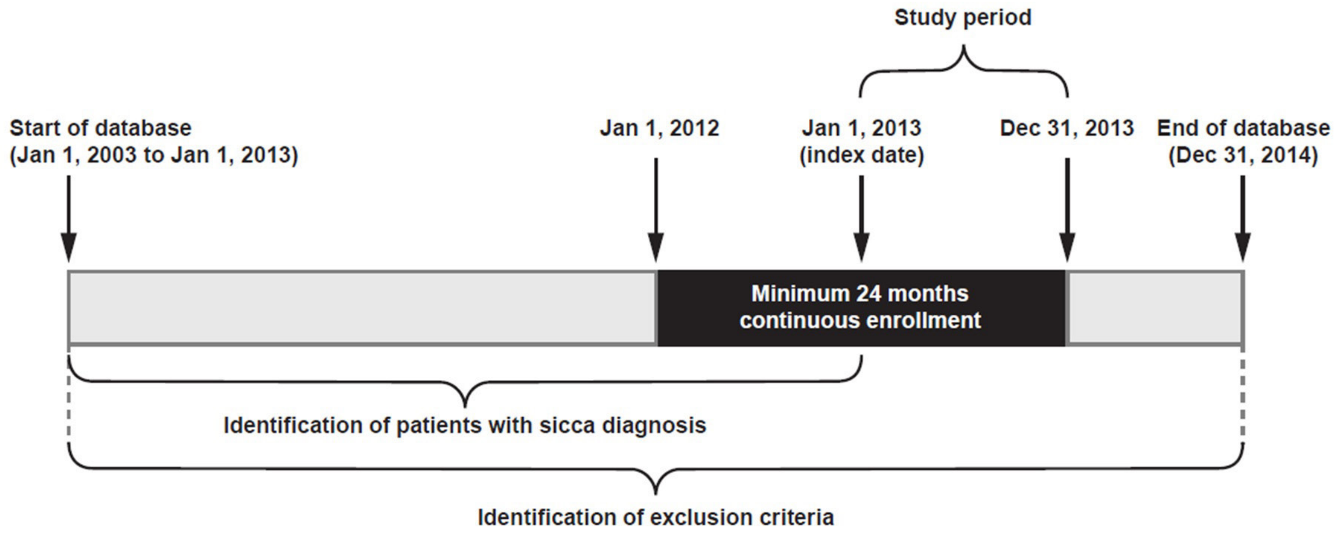

The study population comprised all patients meeting the inclusion criteria, capturing the pool of patients for 2013 (the most recent year for which complete claims data were available). An 'incident cohort' was also assessed, which met the same eligibility criteria and, in addition, were enrolled with a minimum period of 12 months prior to their first recorded sicca diagnosis (to ensure a pSS disease-free period). For simplicity, data from the incident cohort are not presented here. A matched comparator population was included in order to understand the difference in disease burden between patients with sicca and sicca-free subjects. The comparator population comprised subjects who did not have a sicca diagnosis at any point, and were matched to patients with pSS by year of birth, gender, month and year of enrollment, duration of time continuously enrolled, and plan type. A 1:1 ratio of patients with sicca and matched comparator subjects was obtained.

### Data analysis

Descriptive statistics (mean, frequency) were used to describe patient demographic characteristics while the number of patients with glandular versus extra glandular disease were reported to describe clinical characteristics of interest. Extra-glandular disease was defined as a patient having any one or more of the following nine symptom categories: lymphoma, joint, cutaneous, lung, renal, muscular, peripheral nervous system, central nervous system and hematological involvement. The most common comorbidities and medications were also described using descriptive statistics and reported for patients with glandular versus extra-glandular disease. Healthcare resource use and cost burden involved in treating patients with pSS were assessed by extracting information relating to inpatient, outpatient, and emergency care use, and prescriptions. The resource type was categorized as Sjögren's syndrome related (SS related) if patients had a primary diagnosis of sicca syndrome or any of the nine extra-glandular disease manifestations of interest. No inferential statistical analysis was undertaken for the cohorts as the study was intended to be descriptive in nature only.

## RESULTS

### Patient demographics and clinical characteristics

Of 119 899 484 subjects who had pharmacy coverage within the Truven MarketScan Commercial database, 12 717 patients met the eligibility criteria for the primary cohort. Patients had a mean age of 51 years and were more commonly represented in older age categories, with the highest representation in the 50–59-year-old group (40.4%) (Figure 2). The majority of patients were female (86.0%). The most frequent comorbidity of interest was hypertension, followed by mood disorders, depression, anxiety, and keratoconjunctivitis sicca. A similar proportion of patients in the primary cohort (26.8%) and the comparator group (25.5%) experienced hypertension. The proportions of patients experiencing other common comorbidities, such as mood disorders and depression, were slightly higher in the primary cohort (mood disorder, 15.9%; depression, 15.0%) than in the comparator population (mood disorder, 10.3%; depression, 9.7%). Analysis of the clinical profile of the primary cohort showed that 60.7% of patients had claims associated with extra-glandular disease manifestations. Patients with extra-glandular disease had a higher disease burden compared with patients with glandular disease only; they more commonly reported pain (14.7% vs 0.6%, respectively), fatigue (13.0% vs 8.7%, respectively), insomnia (2.8% vs 2.2%, respectively), or any combination of pain, fatigue and/or insomnia (41.3% vs 12.4%, respectively; Figure 3).

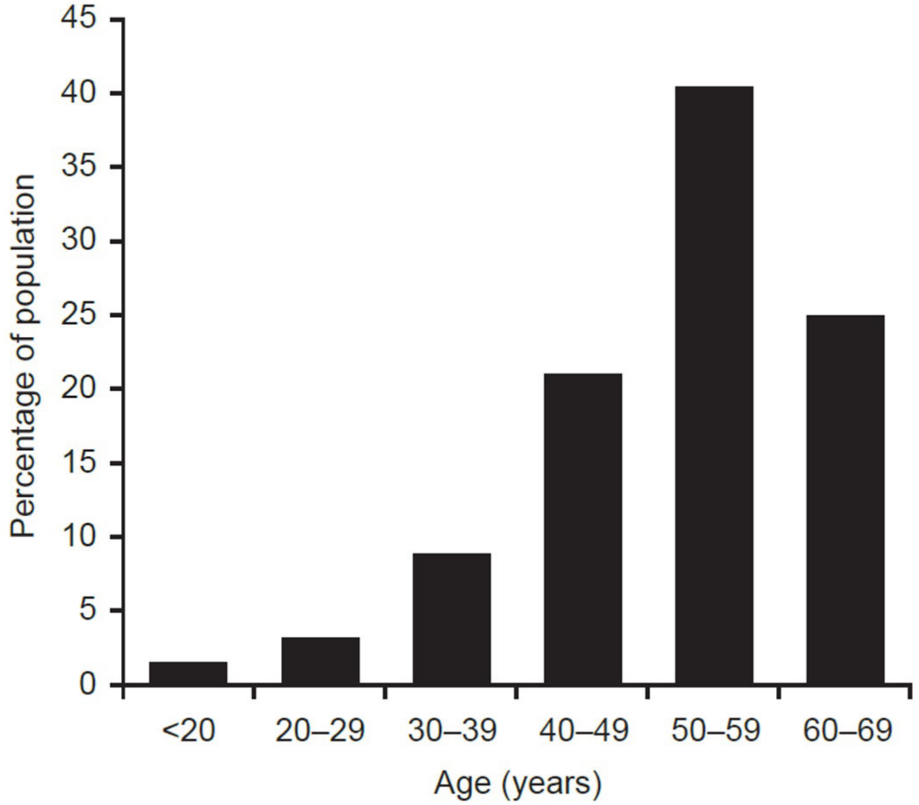

**Figure 2.** Age Distribution of Patients with pSS in the Primary Cohort pSS: primary Sjögren's syndrome

**Figure 3.** Distribution of Clinical Characteristics Reported in Patients with Sicca with A) Glandular Disease or B) Extra-glandular Disease (Primary Cohort)

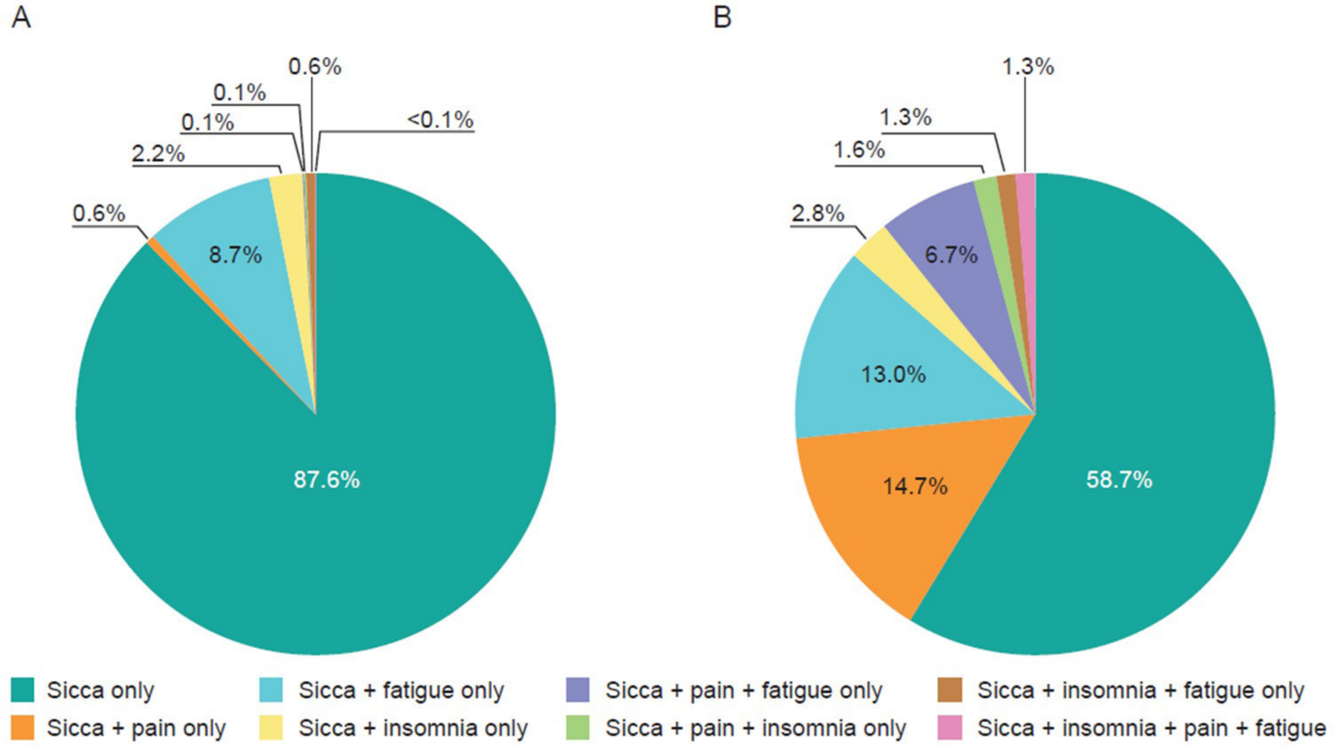

## Treatment patterns and healthcare utilization

*Economic burden*

In the primary cohort, patients incurred a higher cost for healthcare service use compared with the matched comparator group, with the average annual cost per patient being 1.6 times higher for all causes, and 1.5 times higher for SS related causes (Figure 4). When comparing healthcare costs by disease characteristics, patients with extra-glandular disease manifestations incurred a 2.9 times higher average annual cost than those who presented with glandular disease only (Figure 4, Table 1).

**Figure 4.** Economic Burden of the Primary Cohort (Sicca) Versus the Comparator Cohort (Sicca-free)

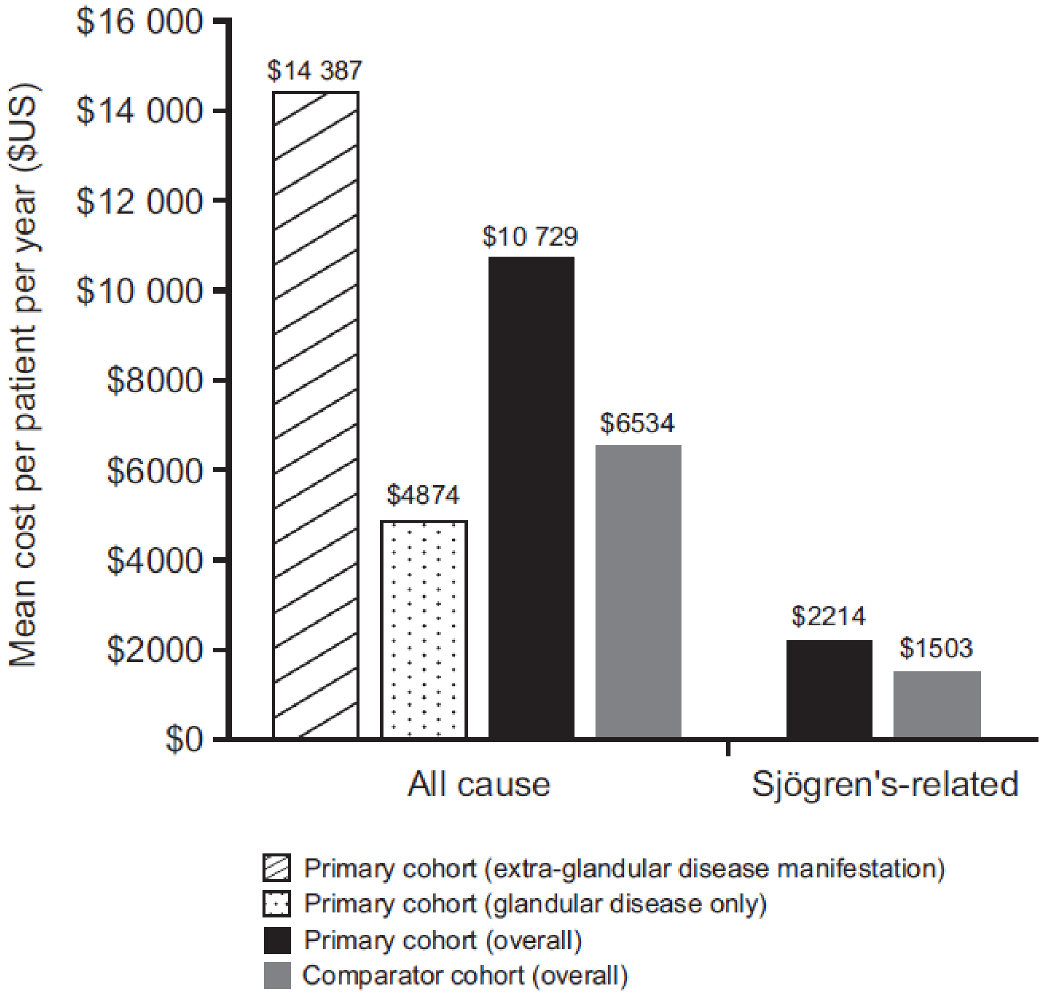

US$ are 2013 value

*Resource utilization*

A greater proportion of the primary cohort used healthcare resources, both all cause and SS-related, compared with the comparator group. The differences were most marked in the number of office visits, with the primary cohort making a visit an average number of 14.6 (all cause) and 5.8 (SS related) times per patient compared with 8.9 (all cause) and 4.7 (SS related) times in the comparator group (Table 2). Within the primary cohort, patients with extra-glandular disease manifestations had a higher healthcare resource use than patients with glandular disease only. Patients with extra glandular disease manifestations had more than double the resource use for outpatient services per year (Table 1).

*Medication use*

Analysis of the pre-defined medications of interest (typical first, second or third-line medications used to treat pSS) showed that the most common medications used were prednisone (14.0%), hydroxychloroquine (12.3%), and cyclosporine (11.6%) (Table 3). The most frequently prescribed medication in the primary cohort was codeine, with 28.4% of all patients having a prescription (Table 4). When comparing medication use across clinical characteristics, there was a general trend for increased medication use with increasing disease burden. Azathioprine was infrequently prescribed in the overall sample; however, it was prescribed in greater frequency in patients with extra-glandular disease manifestations compared with patients with glandular disease only (Table 3).

**Table 1.** Comparison of Healthcare Costs and Resource Use by Disease Characterization (Primary Cohort)

| | Glandular disease only (n=4997) | | Extra-glandular disease manifestations (n=7720) | |
|---|---|---|---|---|
| Mean total cost per patient per year: all services | $4878 (n=4827)[a] | | $14 387 (n=7720) | |
| **Total resource use** | | | | |
| | **Total number of events** | **Average events per patient** | **Total number of events** | **Average events per patient** |
| Office visit | 37 956 | 8.2 | 141 468 | 18.4 |
| Hospital outpatient | 6689 | 2.7 | 29 865 | 5.1 |
| Outpatient (NCE) | 362 | 2.1 | 1103 | 3.8 |
| Hospital inpatient | 136 | 1.1 | 928 | 1.4 |
| Hospital emergency room | 562 | 1.3 | 2831 | 1.7 |
| Urgent care facility | 310 | 1.4 | 847 | 1.7 |

NCE: not classified elsewhere
aOnly patients with non-zero costs are included; US$ are 2013 value

**Table 2.** Healthcare Resource Utilization in the Primary Cohort and Comparator Group

| | Primary cohort (pSS) N=12 717 | | | Comparator group (sicca-free) N=12 717 | | |
|---|---|---|---|---|---|---|
| | Number of patients with at least one event | Total number of events | Average number of events per patient | Number of patients with at least one event | Total number of events | Average number of events per patient |
| **Healthcare utilization (SS related)** | | | | | | |
| **Outpatient services** | | | | | | |
| 1. Office visit | 7696 | 44 376 | 5.8 | 3921 | 18 277 | 4.7 |
| 2. Outpatient hospital | 3013 | 8697 | 2.9 | 1527 | 3964 | 2.6 |
| 3. Outpatient (NCE) | 91 | 127 | 1.4 | 61 | 74 | 1.2 |
| **Inpatient/hospitalizations** | | | | | | |
| 1. Inpatient hospital | 46 | 56 | 1.2 | 37 | 41 | 1.1 |
| **Emergency care** | | | | | | |
| 1. Hospital emergency room | 534 | 651 | 1.2 | 316 | 382 | 1.2 |
| 2. Urgent care facility | 72 | 84 | 1.2 | 55 | 59 | 1.1 |
| **Healthcare utilization (all cause)** | | | | | | |
| **Outpatient services** | | | | | | |
| 1. Office visit | 12 310 | 179 424 | 14.6 | 11 335 | 100 594 | 8.9 |
| 2. Outpatient hospital | 8340 | 36 554 | 4.4 | 6676 | 21 905 | 3.3 |
| 3. Outpatient (NCE) | 465 | 1465 | 3.2 | 404 | 955 | 2.4 |
| **Inpatient/hospitalizations** | | | | | | |
| 1. Inpatient hospital | 792 | 1064 | 1.3 | 614 | 746 | 1.2 |
| **Emergency care** | | | | | | |
| 1. Hospital emergency room | 2122 | 3393 | 1.6 | 1609 | 2322 | 1.4 |
| 2. Urgent care facility | 726 | 1157 | 1.6 | 542 | 736 | 1.4 |

NCE: not classified elsewhere; pSS: primary Sjögren's syndrome; SS: Sjögren's syndrome

**Table 3.** Use of Pre-defined Medications of Interest in the Primary Cohort and by Disease Characterization

| Medication | All patients (N=12 717) | Glandular disease only (n=4997) | | Extra-glandular disease manifestations (n=7720) |
|---|---|---|---|---|
| | | Sicca only: no pain, no fatigue (n=4489) | Sicca + pain or fatigue (n=508) | Sicca +/- pain or fatigue (n=7720) |
| | n (%) | n (%) | n (%) | n (%) |
| Prednisone | 1774 (14.0) | 371 (8.3) | 60 (11.8) | 1343 (17.4) |
| Hydroxychloroquine | 1568 (12.3) | 392 (8.7) | 69 (13.6) | 1107 (14.3) |
| Cyclosporine | 1472 (11.6) | 444 (9.9) | 76 (15.0) | 952 (12.3) |
| Methylprednisolone | 1172 (9.2) | 231 (5.2) | 29 (5.7) | 912 (11.8) |
| Dexamethasone | 137 (1.1) | 26 (0.6) | 4 (0.8) | 107 (1.4) |
| Hydrocortisone | 31 (0.2) | 3 (0.1) | 3 (0.6) | 25 (0.3) |
| Prednisolone | 15 (0.1) | 8 (0.2) | 0 (0) | 7 (0.1) |
| Azathioprine | 91 (0.7) | 17 (0.4) | 1 (0.2) | 73 (1.0) |
| Methotrexate | 147 (1.2) | 21 (0.5) | 4 (0.8) | 122 (1.6) |
| Mycophenolate | 67 (0.5) | 8 (0.2) | 0 (0) | 59 (0.8) |

**Table 4.** Top Ten Most Common Medication Classes in the Primary Cohort and by Disease Characterization

| Top 10 most common medication classes | All patients (N=12 717) | Glandular disease only (n=4997) | | | | Extra-glandular disease manifestations (n=7720) |
|---|---|---|---|---|---|---|
| | | Sicca only: no pain, no fatigue (n=4489) | Sicca + pain (n=36) | Sicca + fatigue (n=465) | Sicca + pain + fatigue (n=7) | Sicca +/- pain or fatigue (n=7720) |
| Codeine and combination, non injectable | 3606 (28.4) | 709 (15.8) | 16 (44.4) | 89 (19.1) | 1 (14.3) | 2791 (36.2) |
| Anti-arthritic, plain | 2941 (23.1) | 578 (12.9) | 5 (13.9) | 73 (15.7) | 0 (0) | 2285 (29.6) |
| Proton pump inhibitor | 2917 (22.9) | 658 (14.7) | 8 (22.2) | 96 (20.7) | 4 (57.1) | 2151 (27.9) |
| Extended spectrum macrolide | 2773 (21.8) | 765 (17.0) | 8 (22.2) | 96 (20.7) | 0 (0) | 1904 (24.7) |
| Hormones, cortisone plain, oral | 2753 (21.7) | 588 (13.1) | 9 (25.0) | 77 (16.6) | 1 (14.3) | 2078 (26.9) |
| Synthetic thyroid hormone | 2482 (19.5) | 691 (15.4) | 5 (13.9) | 102 (21.9) | 0 (0) | 1684 (21.8) |
| Inhaled nasal steroids | 2467 (19.4) | 648 (14.4) | 10 (27.8) | 106 (22.8) | 2 (28.6) 1 | 701 (22.0) |
| HMG-CoA reductase inhibitor | 2288 (18.0) | 688 (15.3) | 6 (16.7) | 77 (16.6) | 3 (42.9) | 1514 (19.6) |
| Seizure disorders | 2284 (18.0) | 313 (7.0) | 10 (27.8) | 53 (11.4) | 2 (28.6) | 1906 (24.7) |
| Systemic quinolones | 2195 (17.3) | 525 (11.7) | 7 (19.4) | 74 (15.9) | 0 (0) | 1589 (20.6) |

HMG-CoA, 3-hydroxy-3-methyl-glutaryl-coenzyme A

## DISCUSSION

There is limited information available describing the overall disease burden of pSS. This study characterized a population of patients with pSS based on sicca diagnoses and complex inclusion/exclusion criteria, and described their clinical characteristics and healthcare utilization, using claims based data from the US Truven MarketScan Commercial database. Truven databases include some of the largest claims databases in the US, providing employer-based patient data reflecting real-world treatment patterns and costs. The demographic data in this present study are consistent with published epidemiology; 40.4% of patients were 50–59 years of age and 86.0% of the study population were women. Published epidemiology data for pSS indicate that the mean age of onset is usually in the fourth to fifth decade, with female to male ratios ranging from 20:1 to 9:1.[15,21]

The healthcare utilization data show that patients in the primary cohort incurred a higher treatment cost and used more healthcare resources than the sicca-free matched comparator group. Patients with extra-glandular disease manifestations had a higher symptom burden in terms of reporting pain, fatigue or insomnia, or combinations of these symptoms, which is likely to contribute to the higher healthcare cost incurred compared with patients with glandular disease only. Data on medication use show that most patients with pSS are managed with codeine, and typical first-line treatments such as prednisone, hydroxychloroquine, and methylprednisolone. A drawback of the analysis is the inability to determine the cause for these prescriptions; therefore, it is likely that at least some of these medications were prescribed for reasons unrelated to pSS management. Analysis of comorbidities showed that mental health/psychological and 'dryness' type conditions were predominant in the primary cohort; it is worthwhile noting that codeine use could also lead to oral dryness symptoms, which confounds the interpretation.

A previous study has utilized the Truven databases from an earlier period (January 1, 2006 to December 31, 2011) to compare healthcare resource utilization for 12-month periods pre- and post-diagnosis, in 10 414 patients newly diagnosed with SS.[20] The baseline demographics of this cohort were similar to those of the primary cohort in this study; however, patients with other connective tissue diseases were not excluded. In agreement with our study, the previous study demonstrated that SS has a high economic burden due to both healthcare resource utilization and medication use. Of note, this previous study reported greater resource utilization, with 99% of patients having at least one outpatient visit (mean 26.9 visits) in the 12-month period post-diagnosis, and mean all cause healthcare costs of US$20 416 per patient year (2014 $) compared with US$10 729 (2013 $) reported in the present study.20 This higher resource utilization may be due to the differing inclusion criteria between studies.

Although reasonable sample sizes were available for the study cohorts, a number of limitations should be considered when interpreting the results. Accurate identification of patients with pSS is challenging. No single ICD-9 code exists for pSS, therefore a complex set of inclusion and exclusion criteria were used to determine a diagnosis of the disease. The comparator group was defined as sicca-free, using the same exclusion criteria; however, they may still have claimed for any of the nine extra-glandular disease manifestations, and therefore may have been utilizing disproportionately higher healthcare resources than a typical disease-free population. In addition, medical history outside of the enrollment period was not captured. When evaluating the demographic results, it is important to note that the Commercial database has an age structure that captures patients of working age only, as well as their dependents (thus excluding retirees). Conclusions on medication use were inferred from prescription claims and therefore did not capture information regarding adherence or discontinuation. Furthermore, information for over-the-counter medication was not collected. This study was not designed to include inferential statistical analysis; our study conclusion is based on the descriptive analysis which described the pattern of treatment, cost and healthcare utilization from real-world data.

## CONCLUSIONS

Despite treatment, patients with pSS still experience a high disease burden. Compared with a matched sicca-free cohort, patients with pSS are more costly to treat and use more healthcare resources. Furthermore, patients with extra-glandular disease have a higher symptom burden in terms of pain and fatigue, as well as higher healthcare costs than patients with glandular disease only. This study provides descriptive information on the characteristics and treatment pathways of patients with pSS that may be useful for generating hypotheses to inform future clinical trial designs. Additional studies to further analyze medications, outcomes, disease progression, and extra glandular disease manifestations will be important to expand understanding of unmet needs in pSS.

## ACKNOWLEDGMENTS

This study was funded by GlaxoSmithKline (GSK; study number: HO-15-16077). Jennie Frain, PhD, and Nicole Cash, MRes, PhD, of Fishawack Indicia Ltd, UK, provided medical writing support, which was funded by GSK, but did not contribute to the study design, or acquisition, analysis or interpretation of data. The authors would like to thank Yumi Asukai and John Logie for their contributions to the development of the study protocol and interpretation of the results.

## DISCLOSURES

SP, LM, RP and SR are employees of GSK and hold shares in the company.
